# CD101, a Novel Echinocandin, Possesses Potent Antibiofilm Activity against Early and Mature Candida albicans Biofilms

**DOI:** 10.1128/AAC.01750-17

**Published:** 2018-01-25

**Authors:** Jyotsna Chandra, Mahmoud A. Ghannoum

**Affiliations:** a Center for Medical Mycology, Case Western Reserve University, University Hospitals Cleveland Medical Center, Cleveland, Ohio, USA

**Keywords:** Candida albicans, CD101, echinocandin, biofilm

## Abstract

Currently available echinocandins are generally effective against Candida biofilms, but the recent emergence of resistance has underscored the importance of developing new antifungal agents that are effective against biofilms. CD101 is a long-acting novel echinocandin with distinctive pharmacokinetic properties and improved stability and safety relative to other drugs in the same class. CD101 is currently being evaluated as a once-weekly intravenous (i.v.) infusion for the treatment of candidemia and invasive candidiasis. In this study, we determined (i) the effect of CD101 against early and mature phase biofilms formed by C. albicans *in vitro* and (ii) the temporal effect of CD101 on the formation of biofilms using time-lapse microscopy (TLM). Early- or mature-phase biofilms were formed on silicone elastomer discs and were exposed to the test compounds for 24 h and quantified by measuring their metabolic activity. Separate batches were observed under a confocal microscope or used to capture TLM images from 0 to 16 h. Measurements of their metabolic activity showed that CD101 (0.25 or 1 μg/ml) significantly prevented adhesion-phase cells from developing into mature biofilms (*P* = 0.0062 or 0.0064, respectively) and eradicated preformed mature biofilms (*P* = 0.04 or 0.01, respectively) compared to those of untreated controls. Confocal microscopy showed significant reductions in biofilm thicknesses for both early and mature phases (*P* < 0.05). TLM showed that CD101 stopped the growth of adhesion- and early-phase biofilms within minutes. CD101-treated hyphae failed to grow into mature biofilms. These results suggest that CD101 may be effective in the prevention and treatment of biofilm-associated nosocomial infections.

## INTRODUCTION

Catheter-related infections are a major cause of morbidity and mortality among hospitalized patients and are commonly associated with microbial biofilms formed on catheter surfaces (1). Biofilms are complex three-dimensional structures formed by microorganisms encased within an extracellular matrix comprising proteins, nucleic acids, and polysaccharides (2, 3). During biofilm formation, microbes potentially go through cycles of active shedding (or detachment) that may cause recurring infections at distant sites, despite treatment with multiple courses of antimicrobials (4). Fungi, particularly Candida spp., have been shown to form biofilms on biomaterials (intravascular catheters, dentures, heart valves, implanted devices, contact lenses, etc.), creating a nidus of organism dissemination, which is often associated with persistent infections (3, 5–7).

Different Candida spp., including Candida albicans (the most prevalent and studied species), Candida parapsilosis, Candida tropicalis, and Candida glabrata, are able to form biofilms. Recently, we showed that the emerging multidrug-resistant Candida auris can also form biofilms (8). A hallmark of biofilm formation is the resistance to antimicrobial agents, particularly triazoles (9, 10). Our group earlier showed that the underlying mechanism of triazole resistance in biofilms is phase dependent, with efflux pumps conferring resistance at an early phase and alterations in sterol levels responsible for resistance at intermediate and mature phases (11). The recent emergence of echinocandin resistance (12, 13) has underscored the importance of identifying and developing new antifungal agents that are effective against biofilms.

CD101 is a long-acting novel echinocandin with distinctive pharmacokinetic (PK) properties and improved stability and safety relative to other drugs in the same class (14–17). A phase 2 study is under way, evaluating CD101 as a once-weekly intravenous (i.v.) infusion for the treatment of candidemia and invasive candidiasis. In the current study, we (i) determined the effect of CD101 against early- and mature-phase biofilms formed by C. albicans *in vitro* and (ii) evaluated the temporal effect of CD101 on the formation of biofilms using real-time time-lapse microscopy (TLM). CD101 also has a modified structure that confers both distinctive pharmacokinetic (PK) properties and an improved safety profile relative to other drugs in the same class (18–21). CD101 possesses a long half-life (20, 21) and demonstrates a prolonged efficacy with a wide safety margin (19, 20).

## RESULTS

### CD101 prevents biofilm formation by C. albicans.

**FIG 1:**
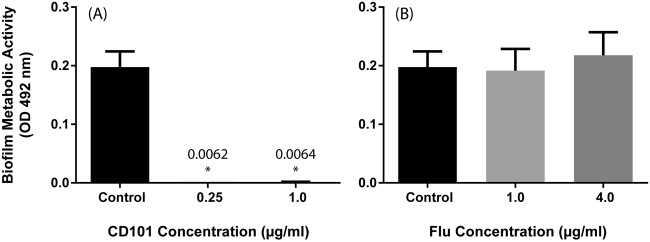
Effects of CD101 and fluconazole on adhesion-phase C. albicans biofilms (prevention). Effects of CD101 (0.25 or 1 μg/ml) (A) and fluconazole (1 or 4 μg/ml) (B) on the metabolic activity of C. albicans biofilms compared to that of the untreated controls. *, *P* value for comparison with the untreated control.

As can be seen in Fig. 1A, the exposure of early-phase C. albicans biofilms to CD101 (0.25 or 1 μg/ml) significantly prevented this pathogen from developing into fully formed biofilms (*P* = 0.0062 or 0.0064, respectively). In contrast, fluconazole failed to inhibit the biofilm formation of C. albicans at both concentrations tested (1 and 4 μg/ml; *P* > 0.05) (Fig. 1B).

**FIG 2:**
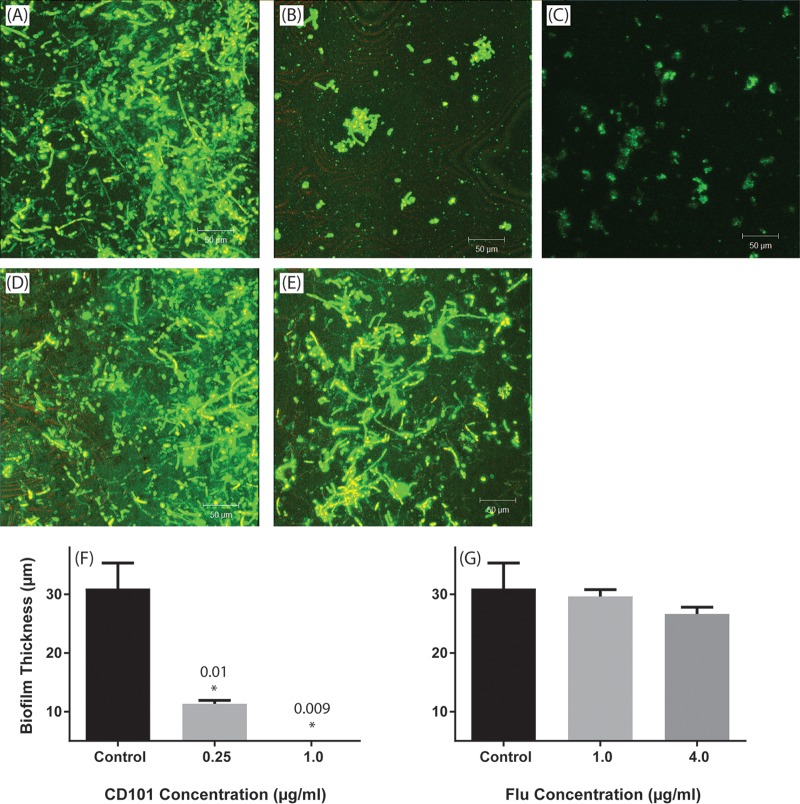
Confocal laser scanning micrographs showing the effects of CD101 and fluconazole on adhesion-phase C. albicans biofilms. Top-down three-dimensional views of biofilms formed by C. albicans treated with no drug (control) (A), 0.25 μg/ml CD101 (B), 1 μg/ml CD101 (C), 1 μg/ml fluconazole (D), or 4 μg/ml fluconazole (E). Thicknesses of Candida biofilms exposed to CD101 (F) or fluconazole (G). *, *P* value for comparison with the untreated control.

Confocal laser scanning microscopy (CLSM) images of untreated control biofilms showed a heterogeneous architecture of biofilms comprising cells/hyphae embedded within the extracellular matrix (Fig. 2A). In contrast, C. albicans cells exposed to both concentrations of CD101 showed only remnants of adhered fungal cells which failed to develop into mature biofilms (Fig. 2B and C). As expected, fluconazole treatment did not inhibit biofilm formation (Fig. 2D and E). The exposure to CD101 significantly reduced biofilm thickness compared to that of untreated control (36 μm versus 4 μm; *P* < 0.05) (Fig. 2F), while fluconazole had no effect on biofilm thickness (Fig. 2G).

### CD101 is effective in eradicating mature-phase biofilms.

**FIG 3:**
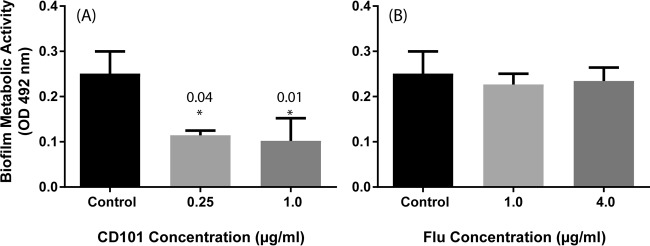
Effects of CD101 and fluconazole on mature-phase C. albicans biofilms (treatment). Effects of CD101 (0.25 or 1 μg/ml) (A) and fluconazole (1 or 4 μg/ml) (B) on metabolic activity of C. albicans biofilms compared to that of the untreated controls. *,*P* value for comparison with untreated control.

**FIG 4:**
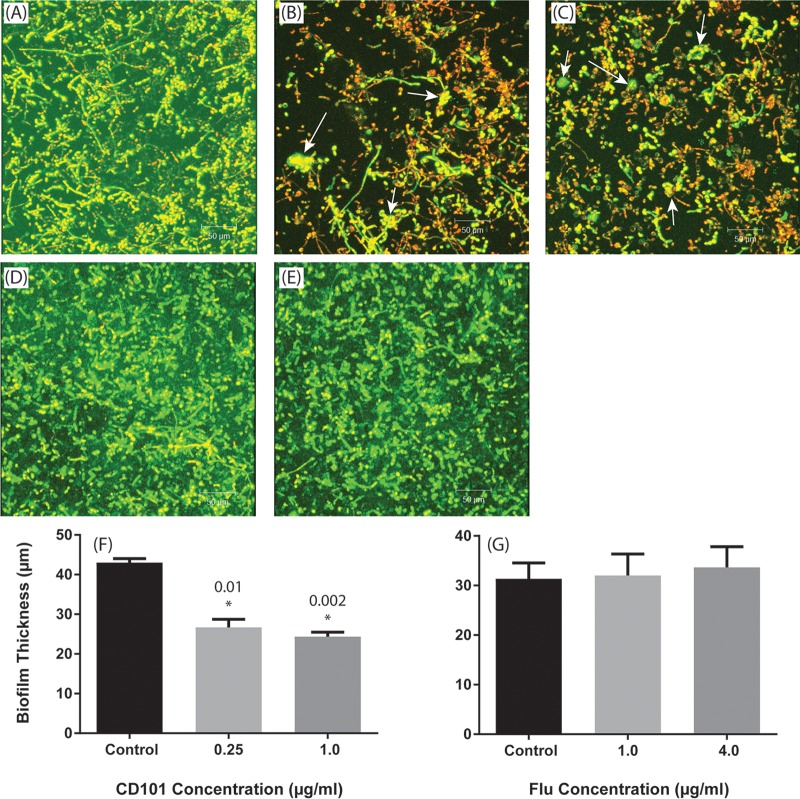
Confocal laser scanning micrographs showing the effects of CD101 and fluconazole on mature-phase C. albicans biofilms. Top-down three-dimensional views of biofilms exposed to no drug (A), 0.25 μg/ml CD101 (B), 1 μg/ml CD101 (C), 1 μg/ml fluconazole (D), or 4 μg/ml fluconazole (E). Arrows show bulged/broken cells. Thicknesses of Candida biofilms exposed to CD101 (F) or fluconazole (G). *, *P* value for comparison with untreated control.

Mature C. albicans biofilms exposed to CD101 (0.25 and 1 μg/ml) exhibited significantly less metabolic activity compared to that of untreated biofilms (*P* = 0.04 and 0.01, respectively) (Fig. 3A). In contrast, neither concentration of fluconazole (1 or 4 μg/ml) affected these biofilms (*P* > 0.05 compared to untreated controls) (Fig. 3B). CLSM analyses showed that untreated control C. albicans biofilms had highly heterogeneous biofilm architectures (Fig. 4A), while biofilms treated with CD101 were eradicated and showed deformed/broken cells (Fig. 4B and C). In contrast, fluconazole did not affect Candida biofilms at either of the concentrations tested (Fig. 4D and E). Additionally, CD101 significantly reduced the thickness of biofilms compared to that of the untreated control (43 μm versus 24 μm; *P* < 0.05) (Fig. 4F), while fluconazole had no effect on biofilm thickness (37 μm versus 36 μm for untreated control and fluconazole, respectively; *P* > 0.05) (Fig. 4G).

### Temporal effect of CD101 on the ability of C. albicans to form biofilms.

**FIG 5:**
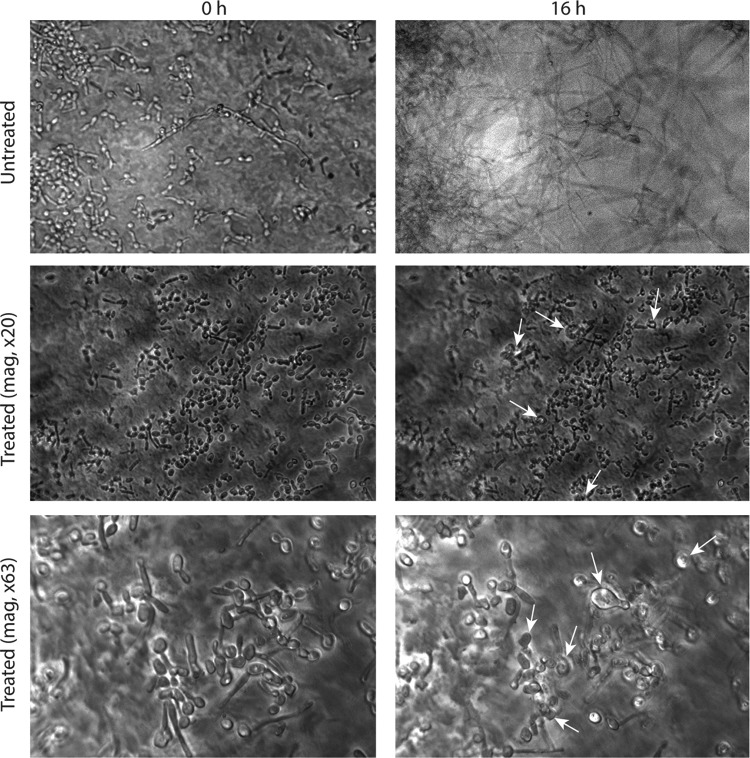
Temporal effect of CD101 (0.25 μg/ml) on formation of C. albicans biofilms. Images were captured immediately (at 0 h) and after 16 h for untreated biofilms and those treated with 0.25 μg/ml CD101 (at ×20 and ×63 magnifications). Arrows show bulging, deformed, and broken cells. The time-lapse movies of the effect of CD101 on C. albicans biofilms and of the untreated control can be seen in Movies S1, S2, and S3 in the supplemental material.

Captured time-lapse movie images showed that untreated C. albicans blastospores adhering to catheter discs started to germinate within 2 h. These germ tubes extended and formed a mesh of hyphae within 3 h. After 6 h, the biofilm matrix started to engulf the fungal structures and by 10 h, a highly heterogeneous 3-dimensional architectural biofilm structure was formed, which consisted of yeast and hyphae embedded within an extracellular matrix, representing a mature biofilm (Fig. 5A, screen frames; see also the time-lapse movie of untreated controls in Movie S1 in the supplemental material). In contrast, germinating fungal cells exposed to 0.25 μg/ml CD101 were arrested in their development, where they showed only adhered cells with short hyphae and stunted growth (until 16 h, mature phase). Moreover, under high magnification (×63), bulged/deformed cells were visible after 2 h. CD101 disrupted fungal cells and caused hyphal tips to burst, releasing string-like contents after 9 to 10 h (see Movie S3). Even after incubation for 16 h, CD101-treated hyphae failed to grow into mature biofilms (Fig. 5B and C; see also Movies S2 and S3).

**FIG 6:**
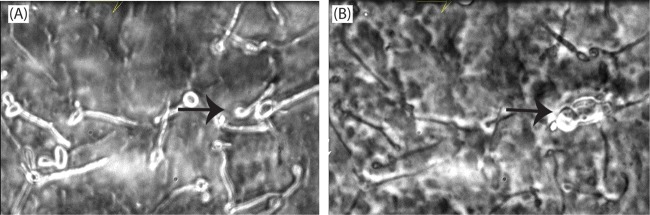
Temporal effect of CD101 (0.25 μg/ml) on 3-h-formed C. albicans biofilms. Drug was added after 3-h biofilm formation, and images were captured immediately after adding the drug (A) and after 16 h (B) (magnification, ×63). Arrows show bulging, deformed, and broken cells. The time-lapse movie of the effect of CD101 on 3-h-formed C. albicans biofilms can be seen in Movie S4.

In other experiments, C. albicans blastospores were allowed to germinate and form biofilms for 3 h post adherence before adding CD101 (0.25 μg/ml). Time-lapse photographic images were captured immediately after adding the drug and for up to 16 h. As expected, the untreated control germinating cells progressed toward forming mature biofilms in a timely manner, with a mature C. albicans biofilm clearly observed by 16 h (data not shown). In contrast, the growth of 3-h-old germinating fungal biofilm cells was stopped within minutes after they were exposed to 0.25 μg/ml CD101 (Fig. 6A and B; see also Movie S4). Moreover, under high magnification, bulged/deformed hyphae were visible after 3 h, and broken cells/hyphae with their tips bursting and releasing contents from them were visible after 8 to 10 h (Fig. 6A and B, arrows; see also Movie S4). By 16 h, CD101-treated hyphae failed to grow into mature biofilms (Fig. 6A and B; see also Movie S4).

## DISCUSSION

Our data demonstrate that CD101 possesses antibiofilm activity against both adhesion-phase and mature-phase biofilms formed by C. albicans, indicating that this drug may be effective in both the prevention and the treatment of Candida biofilms.

Our finding that CD101 possesses antibiofilm activity is consistent with previous studies of echinocandins published by our group showing that, unlike triazoles (fluconazole and voriconazole) and conventional amphotericin B, caspofungin and micafungin had potent antibiofilm activities (10). Others have also shown inhibitory effects of echinocandins against planktonic and biofilm forms of Candida species. Marcos-Zambrano et al. (22) compared the antifungal activities of micafungin and amphotericin B against C. tropicalis and showed that micafungin was most effective against C. tropicalis biofilms. These investigators also showed that the exposure to micafungin promoted significant alterations in biofilm structure, with a reduction in the number of hyphae and an appearance of swollen blastospores (22). In another study, Renátó Kovács et al. (23) also reported that micafungin and caspofungin were active against Candida biofilms *in vitro*, with MICs ranging between 32 to 256 μg/ml and 16 to 512 μg/ml, respectively (23). In our study, we found that CD101 was effective against Candida biofilms at concentrations of 0.25 μg/ml, with drastic effects on the morphology and architecture of biofilms (the remnants of adhered bulged deformed/broken cells with hyphal tips that had burst and released their contents were observed).

To understand the process of Candida biofilm development and the effects of antifungal agents on biofilms, Kaneko et al. (24) analyzed real-time data comprising time-lapse images taken at times separated by brief intervals. For the antifungal study, they treated 5-h-old biofilms of C. albicans with either micafungin or fluconazole. Micafungin began to suppress biofilm growth a few minutes after the initiation of the treatment, and this effect was maintained over the course of the observation period (24). Our results in the present study of 3-h-old germinating biofilm cells showed that CD101 began to suppress biofilm formation a few minutes after treatment initiation and was maintained over the course of the 16 h experiment. Kaneko et al. (24) also showed that micafungin was involved in the disruption of cells in the biofilms, releasing string-like structures (undefined extracellular components) from the burst hyphae. Thus, micafungin acted immediately and suppressed biofilm growth. In our study, CD101 caused disruption/deformation of adhering cells and 3-h-old germinating biofilm cells, releasing cytoplasmic contents from them and preventing cells from developing into a mature biofilm.

Earlier studies have shown that CD101 has a modified structure that confers both distinctive pharmacokinetic (PK) properties and an improved safety profile relative to other drugs in the same class (19–21). CD101 possesses a long half-life (20, 21) and demonstrates a prolonged efficacy with a wide safety margin (19, 20). Moreover, the high plasma drug exposure of CD101 (area under the concentration-time curve from 0 to 168 h [AUC_0–168_] of 813 μg · h/ml for 200 mg and 1,840 μg · h/ml for 400 mg) (17) and its distribution to sites of infection are promising (25, 26). With an extended weekly dosing, CD101 showed better prophylactic and treatment efficacies for invasive candidiasis than micafungin (25). In a comparative study in mice infected by a C. albicans strain, CD101 demonstrated earlier and faster burden reduction than micafungin (25). The MICs of CD101 are comparable to those of the clinically available echinocandins (15, 16). CD101 also has demonstrated potent *in vitro* activities against a broad range of Candida species, including some antifungal-resistant strains (27, 28). The combination of potent *in vitro* activity and enhanced *in vivo* exposure/distribution properties could help to reduce the potential for resistance development during the course of therapy, beyond the low potential observed *in vitro* (29), and to enable the treatment of some echinocandin-resistant strains, as was demonstrated in a C. glabrata mouse model using a Fks2 hot spot 1 mutant strain (F659V) (30).

The potential of CD101 in the treatment and prevention of biofilms is supported by its mechanism of action and its ability to achieve high drug exposures. Previous studies have shown that β-1,3 glucan constitutes approximately 30 to 60% of the fungal cell wall and is an important component in maintaining the strength of it. Echinocandins bind to the Fks p subunit of the enzyme β-1,3 glucan synthase and block it, preventing the formation of β-1,3 glucan (31, 32). In addition to the production of β-1,3 glucan in fungal cell walls, glucan synthase has been shown to be responsible for the glucan found in the adhesive carbohydrate-rich matrix of Candida biofilms that prevents antifungal drugs from reaching their target and enables biofilm cells to survive high exposures to the drug (33, 34). Notably, in our study, CD101 was able to eradicate mature Candida biofilms *in vitro*, while also being able to prevent adhesion-phase cells from developing into mature biofilms. The higher and longer exposures achieved by CD101 *in vivo* should further amplify any advantages observed *in vitro*. This would have important advantages in preventing biofilm-associated nosocomial infections.

## MATERIALS AND METHODS

### Culture media and test compounds.

Yeast nitrogen base (YNB) and Sabouraud dextrose agar (SDA) media were purchased from Becton Dickinson and Company (Sparks, MD). CD101 powder was provided by Cidara Therapeutics (San Diego, CA), while fluconazole, which was used as a comparator, was purchased from Sigma-Aldrich (St. Louis, MO). The drug stock solutions were reconstituted in dimethyl sulfoxide (DMSO) (CD101) or YNB (fluconazole). The drugs were serially diluted in YNB to achieve the final working concentrations of 0.25 and 1 μg/ml for CD101 and 1 and 4 μg/ml for fluconazole. These concentrations were selected for testing the activity of CD101 or fluconazole against Candida biofilms on the basis of MIC data for CD101 or fluconazole (0.25 or 0.5 μg/ml, respectively), performed according to the CLSI M27-A3 method. We elected to use 4 μg/ml to test activity of fluconazole against Candida biofilms on the basis of previous studies of fluconazole that showed it has a poor antibiofilm activity (2, 10). YNB with no drug and DMSO controls were prepared in parallel and used as a growth control for all experiments. Antifungal agents (powder and reconstituted solution) were stored at −80°C until use.

### Fungal strain.

C. albicans SC5314, a clinical strain obtained from a patient with disseminated candidiasis, was used in the current study.

### Effect of CD101 against Candida biofilms.

The effects of CD101 and fluconazole on adhesion-phase biofilms (representing the prevention of biofilms) and mature-phase biofilms (representing the treatment of biofilms) were determined.

For the evaluation of the ability of CD101 to prevent biofilms, Candida cells were adhered to silicone elastomer (SE) catheter discs, using our catheter-associated biofilm model, for 90 min at 37°C as described previously (2, 3). Next, the discs were incubated for 24 h with CD101 (0.25 or 1 μg/ml concentrations), and cells were allowed to form biofilms at 37°C. To evaluate the ability of CD101 to treat biofilms, separate batches after the adhesion phase were transferred to fresh YNB medium and incubated for a further 24 h at 37°C in the absence of drugs to allow biofilm maturation. Next, mature biofilms were exposed to CD101 (0.25 or 1 μg/ml concentrations) for another 24 h at 37°C. The discs incubated with fluconazole (1 or 4 μg/ml) were used as the comparator, and media-alone discs were used as the control in all experiments.

At the end of the drug exposure, in both the prevention and the treatment experiments, biofilms were quantified by measuring their metabolic activity using a sodium 3′-{1-[(phenylamino)-carbonyl]-3,4-tetrazolium}-bis(4-methoxy-6-nitro)benzene-sulfonic acid hydrate (XTT) assay as described earlier (3). After incubating with the drugs, the discs were transferred to fresh plates containing phosphate-buffered saline (PBS) with XTT and menadione and incubated for 3 h at 37°C, and then the optical density was read at 492 nm. Separate batches of biofilms were transferred to a 12-well plate and incubated for 45 min at 37°C in 4 ml of PBS containing the fluorescent stains FUN 1 (10 μM) and a concanavalin A-Alexa Fluor 488 conjugate ([ConA] 25 μg/ml) as described previously (2, 3). After staining, the discs were observed by CLSM (FUN 1: excitation wavelength, 543 nm; emission wavelength, 560 nm through a long-pass filter; ConA: excitation wavelength, 488 nm; emission wavelength, 505 nm through a long-pass filter) to examine the effects of CD101 on biofilm architecture and thickness (2, 3).

### Temporal effect of CD101 on Candida biofilm formation using TLM.

We used time-lapse microscopy (TLM) to determine the temporal effect of CD101 on the ability of C. albicans to form biofilms. This approach involves the capture of single-frame images at specific intervals in real time, enabling the temporal monitoring of the interactions occurring between the drug and Candida as it forms biofilms. Briefly, following adherence of C. albicans blastospores (yeast forms) to SE for 90 min, the discs were placed in a 35-mm-diameter glass-bottom petri dish (MatTek Corp., Ashland, MA). Next, CD101 (0.25 μg/ml) was added to the petri dish, which was incubated at 37°C to allow for biofilm formation. Phase-contrast images for this interaction were captured in real time over a 16-h period using a Leica DMI 6000 B inverted microscope connected to a Retiga EXi Aqua camera (Q-imaging, Vancouver, British Columbia). To determine the structural changes in the maturing biofilm, the acquisition and analysis of a series of horizontal (*x-y*) optical sections of the biofilm were performed using Metamorph Imaging software (Molecular Devices, Downingtown, PA). A disc with C. albicans blastospores incubated in growth medium alone with no antifungal was used as a control.

### Statistical analyses.

Statistical analyses for all data were performed using GraphPad Prism 6 software. Drug-treated groups were compared to control untreated groups using unpaired *t* tests. A *P* value of < 0.05 was considered significant.

## Supplementary Material

Supplemental material
