# Supplementary material for: CD101, a Novel Echinocandin, Possesses Potent Antibiofilm Activity against Early and Mature Candida albicans Biofilms
Source: Antimicrob Agents Chemother. 2018 Jan 25;62(2):e01750-17. doi: 10.1128/AAC.01750-17 (PMC5786756; doi:10.1128/AAC.01750-17)
Supplement: Supplemental material [file supp_62_2_e01750-17__index.html]

Supplemental material 

# CD101, a Novel Echinocandin, Possesses Potent Antibiofilm Activity against Early and Mature Candida albicans Biofilms

## Supplemental material

- Supplemental file 1 -

  Movie S1

  MP4, 8.6M
- Supplemental file 2 -

  Movie S2

  MP4, 7.9M
- Supplemental file 3 -

  Movie S3

  MP4, 8.5M
- Supplemental file 4 -

  Movie S4

  MP4, 7.6M
- Supplemental file 5 -

  Legends for supplemental movies

  PDF, 68K
