# Supplementary material for: CD101, a Novel Echinocandin, Possesses Potent Antibiofilm Activity against Early and Mature Candida albicans Biofilms
Source: Antimicrob Agents Chemother. 2018 Jan 25;62(2):e01750-17. doi: 10.1128/AAC.01750-17 (PMC5786756; doi:10.1128/AAC.01750-17)
Supplement: Supplemental material [file AAC.01750-17_zac002186844s5.pdf]

SUPPLEMENTAL MOVIE LEGENDS

Movie S1. Temporal Development of *C. albicans* Untreated Mature Biofilms: Time-lapse movie was captured immediately after 0 h and followed upto 16 h. It showed untreated *C. albicans* blastospores adhering to catheter discs at 0 h and started to germinate within 2 h. These germ tubes extended and formed a mesh of hyphae within 3 h. After 6 h, biofilm matrix started to engulf the fungal structures and, by 10 h, a highly heterogeneous 3-dimensional architectural biofilm structure was formed, which consisted of yeast and hyphae embedded within an extracellular matrix representing a mature biofilm.

Movie S2. Temporal Effect of CD101 (0.25 µg/ml) on Formation of *C. albicans* Biofilms (x20): Time-lapse movie was captured immediately after 0 h and followed upto 16 h at low magnification, x20. Adhered fungal cells exposed to 0.25 µg/ml CD101 were arrested in their development and showed only adhered cells with short hyphae and stunted growth (until 16 h, mature phase). Bulged/deformed cells were visible after 2 h (arrows). CD101 disrupted fungal cells and caused hyphal tips to burst releasing string-like contents after 9-10 h (arrows). CD101-treated hyphae failed to grow into mature biofilms.

Movie S3. Temporal Effect of CD101 (0.25 µg/ml) on Formation of *C. albicans* Biofilms (x63) : Time-lapse movie was captured immediately after 0 h and followed upto 16 h at high magnification, x63. Incubation of fungal cells with 0.25 µg/ml CD101 stopped cell growth within minutes after exposure. Adhered cells with short hyphae and stunted growth were visible until 16 h, mature phase (arrows). Bulged/deformed cells were clearly

visible at high magnification, after 2 h (arrows). CD101 disrupted fungal cells and caused hyphal tips to burst releasing string-like contents after 9-10 h, (arrows). CD101-treated hyphae failed to grow into mature biofilms.

Movie S4. Temporal Effect of CD101 (0.25 µg/ml) on 3 h Formed *C. albicans* Biofilms: Time-lapse movie was captured immediately after adding the drug and followed upto 16 h. In this movie, *C. albicans* blastospores were allowed to germinate and form biofilms for 3 h post adherence before adding CD101 (0.25 µg/ml). Growth of 3 h-old germinating fungal biofilm cells was stopped within minutes after they were exposed to 0.25 µg/ml CD101. Cells/hyphae bulged and were deformed after 2-3 h (arrows). Broken hyphae with bursting tips and releasing contents from them were visible after 8-10h (arrows). By 16 h CD101-treated hyphae failed to grow into mature biofilms.
